# Insulin resistance and associated factors among HIV-infected patients in sub-Saharan Africa: a cross sectional study from Cameroon

**DOI:** 10.1186/s12944-017-0543-1

**Published:** 2017-08-10

**Authors:** Steve Raoul Ngongang Noumegni, Jobert Richie Nansseu, Vicky Jocelyne Moor Ama, Jean Joel Bigna, Felix Kembe Assah, Magellan Guewo-Fokeng, Steve Leumi, Jean-Claude Katte, Mesmin Dehayem, Andre Pascal Kengne, Eugene Sobngwi

**Affiliations:** 1 Faculty of Medicine and Biomedical Sciences, University of Yaoundé 1, PO Box 1364, Yaoundé, Cameroon; 2 Faculty of Medicine, University of Paris Sud XI, 63 Av Gabriel Péri, Le Kremlin Bicêtre, France; 3 Department of Public Health, Faculty of Medicine and Biomedical Sciences, University of Yaoundé 1, PO Box 1364, Yaoundé, Cameroon; 4 Department of Biochemistry and Physiological Sciences, Faculty of Medicine and Biomedical Sciences, University of Yaoundé 1, PO Box 1364, Yaoundé, Cameroon; 5 Laboratory of Biochemistry, University Teaching Hospital, Yaoundé, Cameroon; 6 Department of Epidemiology and Public Health, Centre Pasteur of Cameroon, PO Box 1274, Yaoundé, Cameroon; 7 Department of Biochemistry, Faculty of Sciences, University of Yaoundé I, Yaoundé, Cameroon; 8 Laboratory of Molecular Medicine and Metabolism, Biotechnology Center, University of Yaoundé 1, Yaoundé, Cameroon; 9 National Obesity Center, Yaoundé Central Hospital, PO Box 87, Yaoundé, Cameroon; 10 Non-Communicable Diseases Research Unit, South African Medical Research Council, Cape Town, 7505 South Africa; 11 Department of Medicine, Groote Schuur Hospital and University of Cape Town, Cape Town, 8000 South Africa; 12 Departement of Internal Medicine, Faculty of Medicine and Biomedical Sciences, University of Yaoundé 1, PO Box 1364, Yaoundé, Cameroon

**Keywords:** Insulin resistance, HOMA-IR, HIV, Cameroon

## Abstract

**Background:**

Little is known on the magnitude and correlates of insulin resistance in HIV-infected people in Africa. We determined the prevalence of insulin resistance and investigated associated factors in HIV-infected adult Cameroonians.

**Methods:**

We conducted a cross-sectional study at the Yaoundé Central Hospital, Cameroon; during which we enrolled HIV-infected people aged 30 to 74 years with no previous history of cardiovascular disease. An homeostatic model assessment of insulin resistance (HOMA-IR) like index served to assess insulin sensitivity with insulin resistance defined by values of 2.1 or higher.

**Results:**

We included 452 patients (20% men). Their mean age was 44.4 ± 9.8 years and 88.5% of them were on antiretroviral therapy (93.3% on first line regimen including Zidovudine, lamivudine and Efavirenz/Nevirapine). Of all participants, 28.5% were overweight, 19.5% had obesity and 2.0% had diabetes. The prevalence of insulin resistance was 47.3% without any difference between patients on ART and those ART-naïve (48.5% vs. 38.5%; *p* = 0.480). Obesity was the only factor independently associated with insulin resistance (adjusted odds ratio: 2.28; 95% confidence interval: 1.10–4.72).

**Conclusion:**

Insulin resistance is present in nearly half of HIV-infected patients in Cameroon despite a low prevalence rate of diabetes, and is associated with obesity.

## Background

Insulin resistance is a metabolic disorder characterized by a decrease in sensitivity of insulin receptors within target cells, resulting in the failure of insulin to achieve the desired effect [1]. The condition may occur during puberty and pregnancy, in the context of diseases such as obesity, type 2 diabetes, dyslipidemia and ovarian dysfunction, or secondary to continuous use of medications such as steroids [2]. Insulin resistance is associated with high cardiovascular morbidity and mortality [3]. Furthermore, there is growing evidence that insulin resistance can appear in the course of HIV infection, especially after antiretroviral therapy (ART) initiation [4].

Several studies have reported high prevalence of insulin resistance and other metabolic abnormalities in people living with HIV, including hypertriglyceridemia and low levels of high density lipoproteins (HDL-C). These metabolic abnormalities seem to be more preponderant among patients on ART, especially those on protease inhibitors-containing regimen [5, 6]. The prevalence of insulin resistance in HIV-infected individuals varies between 13 and 47.7% [7].

In Africa the epicenter of HIV infection, little is known on the magnitude and correlates of insulin resistance among HIV-infected people. The present study was conducted to determine the prevalence of insulin resistance and associated factors among an HIV-infected African population.

## Methods

### Study design, setting and participants

This was a cross-sectional study with participants recruited between December 2015 and May 2016 at the HIV day-care unit of the Yaoundé Central Hospital, Cameroon. This health care facility is one of the most important HIV clinics in Cameroon, providing specialized care to almost 12,000 HIV-infected persons. The staff of this center includes general practitioners and specialized physicians who are frequently trained in the management of HIV infection. All Blood samples were analyzed at the biochemistry laboratory of the Yaoundé University Teaching Hospital and at the Laboratory of Molecular Medicine and Metabolism, University of Yaoundé 1 Biotechnology Center, Cameroon. The study was approved by the Cameroon National Ethics Committee for Human Health Research (Ethical approval N° 2015/12/710/CE/CNERSH/SP). All consenting clinically stable HIV-infected patients aged 30 to 74 years, with no history of cardiovascular disease and routinely followed at the study site were eligible, unless they were receiving lipid-lowering therapy or hormone therapy, were pregnant or breastfeeding women.

### Data collection

We used a standardized and pre-tested questionnaire to collect data on general characteristics such as age and gender, history of HIV infection including duration since diagnosis, ART regimen and duration, CD4 count of less than 6 months and viral load of less than 1 year, and history of hypertension and/or diabetes.

Weight was measured to the nearest 0.1 kg using an electronic scale (CAMRY, Hong Kong, China); with the participant standing up on the scale without shoes and heavy clothing, looking straight ahead, arms stretching along the body. Height was measured to the nearest 0.1 cm using a stadiometer; with the participant standing up without shoes and hat, looking straight ahead, arms stretching along the body. Body Mass Index (BMI) was then derived as weight (kg)/height*height (m^2^), and participants grouped into 4 categories: underweight (< 18.5), normal (18.5–24.9), overweight (25.0–29.9) or obese (≥30.0) [8]. Waist and hip circumferences were measured to the nearest 0.1 cm using a measuring tape; the waist-to-hip ratio (WHR) was then calculated. Abdominal obesity was defined in accordance with the IDF criteria (waist circumference > 90 cm for men and >80 cm for women) [9]. We used an electronic sphygmomanometer (Omron M5–1, Omron Healthcare, Kyoto, Japan) for blood pressure measurement. Hypertension was defined as a systolic blood pressure (SBP) ≥ 140 mmHg and/or a diastolic blood pressure (DBP) ≥ 90 and/or self-reported history of ongoing antihypertensive medication use [10].

### Blood sampling and laboratory investigations

Blood samples were aseptically collected after a 12-h overnight fasting, by venipuncture of the brachial vein in a 5 ml sodium fluoride tube and a 5 ml dry tube without a tourniquet or fist clenching. Thereafter, they were kept on ice (4 °C) and immediately transported to the biochemistry laboratory where plasma and serum specimen were separated accordingly, by centrifugation at 3.000 rpm for 5 min. Fasting plasma glucose (FPG) and lipids including total cholesterol (TC), triglycerides (TG) and HDL-C were assayed without delay using standard colorimetric methods. Using the Friedwald’s formula, low density lipoproteins cholesterol (LDL-C) was calculated when TG levels were below 4 mmol/L, and measured when TG values were over 4 mmol/L [11]. Serum was aliquoted and stored at −20 °C for further measurement of C-Peptide. C-Peptide was measured within 6 weeks using a previously validated sandwich immunoassay method [12].

Dyslipidemia was considered in the presence of elevated levels of TC (> 6.2 mmol/L) and/or an elevated level of LDL-C (> 4.1 mmol/L) and/or a low HDL-C level (< 1.04 mmol/L in men and 1.29 mmol/L in women) and/or an elevated level of TG (≥ 1.7 mmol/L) [13]. We considered as having diabetes any patient with at least 2 FPG levels ≥7.0 mmol/L on two occasions at least 48 h apart, or self-reported history of antidiabetic medications after the diagnosis was made in a health facility [14]. Metabolic syndrome was defined using the International Diabetes Federation (IDF) criteria [9].

### Determination of Insulin sensitivity

We used an HOMA-IR like index for insulin sensitivity assessment; it was determined by the HOMA-IR formula: FPG (mmol/L) x insulin (mU/L or μUl/mL) / 22.5) [15]; where the insulin level was replaced by the C-peptide level. The C-peptide measurement was use to access insulin level base on their equimolar secretion in blood. In fact, the measurement of insulin would have not be easy according to our facilities; however, we decided to measure the C-peptide because it is co-secreted in equimolar concentration with insulin, it is not metabolized by the liver and does not undergo any extraction during the first hepatic passage [16]. Therefore, it can be indirectly measured to access insulin secretion [16]. Insulin resistance was defined by any value of the HOMA-IR like index equal or above 2.1; this threshold was defined in an HIV-infected population in Peru [7].

### Statistical analysis

Data were analyzed using SPSS v. 23 (IBM Corporation, Chicago, Illinois, USA). Results are presented as count (percentage) for categorical variables, and mean ± standard deviation (SD) or median (25th–75th percentiles) for quantitative variables. The Student t-test, chi-square test or equivalents served for groups’ comparison. Logistic regressions were then used to investigate the determinants of insulin resistance. The basic models included age, sex and each candidate predictor of interest. Expanded multivariable models included age and sex, and significant variables in basic models, based on a threshold *p*-value <0.25 from which backward elimination was used to retain the final independent predictors of insulin resistance. A *p*-value <0.05 was used to characterize statistically significant results.

## Results

### Characteristics of the study population

Four hundred and 52 patients (452) were included, among whom 91 (20.1%) were males. The mean age was 44.4 ± 9.8 years. The duration since diagnosis of HIV infection ranged from 2 days to 204 months with a median of 84 months (25th–75th percentiles: 36–108). Of the 452 included patients, 400 (88.5%) were on ART. Duration of ART varied between 1 and 179 months, with a median of 72 months (25th–75th percentiles: 35–108). Only 6.7% of participants were on second-line treatment, protease inhibitors containing regimen especially; no third line treatment was reported.

BMI, waist circumference and WHR were higher in patients on ART than in ART-naïve ones (Table 1). Obesity and abdominal obesity were significantly more prevalent in ART treated in comparison to their ART-naïve counterparts (Table 1). The mean values of blood pressure were 124 ± 23 mmHg for SBP and 81 ± 14 mmHg for DBP (Table 1).

**Table 1:** Characteristics of the study population

	Overall (*n* = 452)	ART – (*n* = 52)	ART + (*n* = 400)	*p*
Clinical characteristics				
Mean age (years)	44.4 ± 9.8	43.7 ± 9.9	44.7 ± 9.9	0.064
Mean systolic Blood pressure (mmHg)	123.4 ± 22.5	124.0 ± 23.6	123.3 ± 22.4	0.849
Mean diastolic blood pressure (mmHg)	81.3 ± 13.5	82.0 ± 14.7	81.2 ± 13.4	0.700
Hypertension, n (%)	60 (13.3)	11 (21.1)	49 (12.3)	0.075
Mean body mass index (kg/m^2^)	25.8 ± 5.3	24.0 ± 4.8	26.0 ± 5.3	0.006
Obesity, n (%)	218 (48.0)	14 (26.9)	203 (50.8)	0.001
Mean waist circumference (cm)	82.1 ± 11.6	78.1 ± 10.9	82.6 ± 11.6	0.007
Abdominal obesity, n (%)	195 (43.1)	12 (25.0)	182 (45.5)	0.005
Mean hip circumference (cm)	95.1 ± 11.2	90.7 ± 9.5	95.6 ± 11.7	0.001
Mean waist/hip ratio	0.86 ± 0.07	0.86 ± 0.07	0.86 ± 0.07	0.744
Biological characteristics				
Median CD4 count (cells/mm^3^)	375 (245–532)	352 (173–600)	375 (258–523)	0.933
Undetectable Viral load (≤ 60 copies/ml), n (%)	72/84 (85.7)	2/2 (100)	70/82 (85.5)	>0.999
Mean fasting glycaemia (mmol/L)	5.1 ± 0.9	4.9 ± 1.1	5.1 ± 0.9	0.134
Diabetes, n (%)	9 (2.0)	1 (1.9)	8 (2.0)	0.970
Mean total cholesterol (mmol/L)	4.5 ± 1.0	4.0 ± 1.0	4.5 ± 1.0	0.001
Hypercholesterolaemia, n (%)	26 (5.8)	1 (1.9)	25 (6.3)	0.341
Mean HDL-cholesterol (mmol/L)	1.7 ± 0.6	1.2 ± 0.5	1.7 ± 0.6	< 0.001
Low HDL, n (%)	106 (23.5)	28 (55.3)	78 (19.5)	< 0.001
Mean triglycerides (mmol/L)	1.0 ± 0.5	1.1 ± 0.5	1.0 ± 0.5	0.233
Hypertriglyceridaemia, n (%)	35 (7.7)	4 (7.7)	31 (7.8)	0.988
Mean LDLcholesterol (mmol/L)	2.3 ± 0.9	2.3 ± 0.8	2.3 ± 0.9	0.739
High LDL cholesterol, n (%)	17 (3.8)	1 (1.9)	16 (4.0)	0.707
Any dyslipidemia, n (%)	153 (33.8)	30 (57.7)	123 (30.8)	< 0.001
Median C-peptide	9.1 (6.8–13.5)	9.2 (8.0–15.4)	9.1 (6.6–12.6)	0.860
Median HOMA-IR like index	2.0 (1.4–3.2)	1.9 (1.4–2.8)	2.0 (1.4–3.0)	0.219
Insulin resistance, n (%)	214 (47.3)	20 (38.5)	194 (48.5)	0.480
Metabolic syndrome, n (%)	53 (11.7)	3 (5.8)	50 (12.5)	0.156

Values are count (percentages), mean ± standard deviation or median (25th–75th percentiles)

*HDL* high density lipoproteins, *LDL* low density lipoproteins, *ART +* Participants on antiretroviral therapy, *ART-* participants naïve to antiretroviral therapy

Of the 452 participants, 331 (73.9%) had a recent CD4 count of less than 6 months in their files. The values ranged between 2 and 1800 cells/mm^3^ with a median of 375 cells/mm3 (25th–75th percentiles: 245–527) (Table 1).

C-peptide values ranged from 2.4 to 342.6 mU/L, with a median of 9.1 mU/L (25th–75th percentiles: 6.8–13.5) and no difference between ART+ and ART- patients. The mean value of FPG was 5.1 ± 0.9 mmol/L; 9 participants (2.0%) had diabetes (Table 1). The prevalence of dyslipidemia was 33.8%. Overall, 11.7% of participants had metabolic syndrome, with no difference between ART+ and ART- patients (Table 1).

### Insulin resistance and associated factors in the study population

The median HOMA-IR like index was 2.0 (25th–75th percentiles: 1.4–3.2). In all, 214 (47.3%) participants were insulin resistant, with 88.7% of them on ART and 9.9% having diabetes. Diabetes (IR+ vs. IR-: 3.3% vs. 0.8%, *p* = 0.027), obesity (29.0% vs. 10.9%, *p* = 0.006), abdominal obesity (52.3% vs. 34.9%, *p* = 0.025) and to some extent hypertension (17.8% vs. 9.2%, *p* = 0.064) were more frequent in participants with insulin resistance than in those without. In age and sex adjusted analyses, obesity (odds ratio [OR]: 2.50; 95% CI: 1.24–5.06), abdominal obesity (OR: 2.45; 95% CI: 1.03–4.89) and diabetes (OR: 8.96; 95% CI: 1.06–71.21) were significantly associated with insulin resistance (Table 2). In expanded multivariable logistic regression models, general obesity remained the only factor independently associated with insulin resistance (OR: 2.28; 95% CI: 1.10–4.72; *p* = 0.027) (Table 3).

**Table 2:** Factors associated with insulin resistance

Variables	Modalities	IR + (*n* = 214)	IR - (*n* = 238)	Crude odds ratio (95% CI)	*p*	^a^Age and sex adjusted odds ratio (95% CI)	*p*
Gender	- Male	36 (16.8)	55 (23.1)	0.67 (0.37–1.44)	0.355		
	- Female	178 (83.2)	183 (76.9)	1			
Age	- ≥ 46	84 (39.3)	90 (37.8)	1,06 (0.54–3.68)	0.480		
	- < 46	130 (60.7)	148 (62.2)	1			
HIV duration	≥ 12 months	194 (90.7)	224 (94.1)	0.61 (0.32–1.23)	0.613	0.73 (0.29–1.82)	0.500
	< 12 months	20 (9.3)	14 (5.9)	1		1	
ART	- yes	194 (90.7)	206 (86.6)	1.51 (0.54–3.68)	0.480	1.27 (047–3.41)	0.634
	- no	20 (9.3)	32 (13.4)	1		1	
1st line with NNRTIs	- yes	211 (98.6)	162 (68.1)	1.40 (0.76–1.02)	0.372	0.92 (0.30–1.70)	0.457
	- no	13 (1.4)	14 (31.9)	1		1	
2nd line with PIs	- yes	10 (4.7)	17 (7.1)	0.64 (0.15–2.84)	0.722	0.66 (0.15–2.87)	0.576
	- no	179 (95.3)	194 (92.9)	1		1	
Metabolic syndrome	- yes	35 (16.4)	18 (7.6)	2.39 (0.84–4.28)	0.120	1.79 (0.76–4.19)	0.178
	- no	179 (83.6)	220 (92.4)	1		1	
Hypertension	- yes	38 (17.8)	22 (9.2)	2.12 (0.96–3.92)	0.064	1.89 (0.92–3.85)	0.079
	- no	176 (82.2)	216 (90.8)	1		1	
Diabetes	- yes	7 (3.3)	2 (0.8)	4.00 (1.02–71.20)	0.027	8.96 (1.06–76.21)	0.044
	- no	207 (96.7)	236 (99.2)	1		1	
Obesity	- yes	62 (29.0)	26 (10.9)	3.32 (1.30–4.84)	0.006	2.50 (1.24–5.06)	0.011
	- no	152 (71.0)	212 (89.1)	1		1	
Abdominal obesity	- yes	112 (52.3)	83 (34.9)	2.05 (1.17–4.40)	0.025	2.45 (1.03–4.89)	0.041
	- no	102 (47.7)	155 (65.1)	1		1	
Dyslipidemia	- yes	65 (30.4)	88 (37.0)	0.74 (0.37–1.35)	0.286	0.72 (0.37–1.40)	0.337
	- no	149 (69.6)	150 (63.0)	1		1	
CD4 count	- ≥ 350	89 (41.6)	101 (42.4)	1.30 (0.60–2.85)	0.508	1.31 (0.59–2.89)	0.508
	- < 350	57 (58.4)	84 (57.6)	1		1	
Detectable viral load	- yes	8 (3.7)	4 (1.7)	2.50 (0.20–31.80)	0.583	/	/
	- no	32 (96.3)	40 (98.3)	1		1	

^a^Adjusted for: age and gender. Factors are presented as count (percentage)

*ART* antiretroviral therapy, *NNRTIs* non-nucleoside reverse transcriptase inhibitors, *IR +* participants with insulin resistance, *IR-* participants without insulin resistance

**Table 3:** Factors associated with insulin resistance, models with general obesity or abdominal obesity

Variables	Modalities	IR + (*n* = 214)	IR - (*n* = 238)	^a^Adjusted odds ratio (95% CI)	*p*	^b^Adjusted odds ratio (95% CI)	*p*
Gender	- Male	36 (16.8)	55 (23.1)	1.06 (0.49–2.89)	0.888	1.21 (0.52–2.83)	0.660
	- Female	178 (83.2)	183 (76.9)	1		1	
Age	- ≥ 46	84 (39.3)	90 (37.8)	1,31 (0.45–3.79)	0.618	1.45 (0.51–4.16)	0.485
	- < 46	130 (60.7)	148 (62.2)	1			
1st line with NNRTIs	- yes	211 (98.6)	162 (68.1)	0.91 (0.32–2.56)	0.854	0.87 (0.31–2.43)	0.790
	- no	13 (1.4)	14 (31.9)	1		1	
2nd line with PIs	- yes	10 (4.7)	17 (7.1)	0.94 (0.17–5.37)	0.944	0.93 (0.17–5.56)	0.935
	- no	179 (95.3)	194 (92.9)	1		1	
Metabolic syndrome	- yes	35 (16.4)	18 (7.6)	1.10 (0.43–2.81)	0.845	0.98 (0.36–2.70)	0.968
	- no	179 (83.6)	220 (92.4)	1		1	
Hypertension	- yes	38 (17.8)	22 (9.2)	1.91 (0.89–4.08)	0.096	1.87 (0.87–4.01)	0.109
	- no	176 (82.2)	216 (90.8)	1		1	
Diabetes	- yes	7 (3.3)	2 (0.8)	7.34 (0.86–62.74)	0.069	8.36 (0.97–72.10)	0.053
	- no	207 (96.7)	236 (99.2)	1		1	
General obesity	- yes	62 (29.0)	26 (10.9)	2.28 (1.10–4.72)	0.027		
	- no	152 (71.0)	212 (89.1)	1			
Abdominal obesity	- yes	112 (52.3)	83 (34.9)			2.11 (0.95–4.69)	0.068
	- no	102 (47.7)	155 (65.1)			1	

^a^Adjusted for: gender, age, PIs, NNRTIs, obesity, diabetes, hypertension, metabolic syndrome and general obesity.

^b^Adjusted for: gender, age, PIs, NNRTIs, obesity, diabetes, hypertension, metabolic syndrome and abdominal obesity. Factors are presented as count (percentage)

*NNRTIs* non-nucleoside reverse transcriptase inhibitors, *PIs* protease inhibitors, *IR +* participants with insulin resistance, *IR-* participants without insulin resistance

## Discussion

In this study including HIV-infected Cameroonians, we found a high prevalence of insulin resistance, affecting nearly half of participants with no previous cardiovascular disease. In these relatively young patients with insulin resistance, diabetes was frequent, as affecting one in ten participants. Moreover, obesity appeared as an independent factor which explains insulin resistance occurrence in these patients. Preventing and controlling obesity in our HIV-infected population, will likely lead to a reduction or delay in insulin resistance occurrence, as well as related harmful consequences.

The prevalence of insulin resistance in our sample was higher than previously reported by several studies like that of Guillen and co-workers in a large HIV-infected population in Peru; based also on the HOMA-IR like index and applying the same threshold definition [7]. This difference can be explained by the fact that basal insulin secretion seems higher in subjects of African origins compared to others [17]. However, our prevalence is within the range previously described in the literature, that is between 13 and 47.7% [7, 18]. Discrepancies observed between studies may be partly due to the method used to assess insulin sensitivity. In fact, although the general trend is towards using the HOMA-IR index, there is no current consensus on which method should be used for insulin sensitivity evaluation among HIV-infected patients. Moreover, even when using indirect assessment tools like the HOMA-IR index, the main difficulty remains the threshold to consider in defining insulin resistance, since this threshold may vary depending on race, gender and even some pathologies [19].

Diabetes was present in one participant out of ten with insulin resistance. This high prevalence of diabetes in this relatively young population can be explained by the role of insulin resistance as a predicting factor for diabetes [20]. General obesity and abdominal obesity were also frequent in our study population; these high prevalences are in contrast with weight loss frequently reported in HIV-infected populations [21]. These results show how important it is to avoid compensatory over-nutrition in this high risk population.

General obesity remained the only factor associated to insulin resistance after adjusting for other factors in multivariable analysis. These results are similar to those reported by Geffner and co-workers who described general obesity as the factors more closely associated with insulin resistance diagnosed by HOMA-IR index in a cohort of US HIV-infected children and adolescents [22]. Similarly, Guillen and co-workers showed an association between greater body mass index and insulin resistance diagnosed by HOMA-IR index in HIV-infected adults in Peru [7]. On the other hand and in line with Guillen and co-workers’ findings, we did not observe an association between insulin resistance and ART; even when the ART regimen comprised protease inhibitors. These results differ from those previously reported [6, 23]. Moreover, there is convincing evidence that development of insulin resistance and other abnormalities of glucose metabolism in the context of HIV infection can have multiple origins including continuous ART, and inflammatory reactions associated with chronic HIV infection [18, 24].

Our study has some limitations. First, its cross-sectional design does not allow us to conclude on a causative-relationship between insulin resistance and identified associated factors. Second, our sampling method was not random and we recruited our patients from only one site, this can be a source of bias and may hamper the generalization of our findings to the entire Cameroonian HIV-infected population. Third, we used C-peptide measurement to access insulin level base on their equimolar secretion in blood. In fact, the measurement of insulin would have not be easy according to our facilities; however, we decided to measure the C-peptide because it is co-secreted in equimolar concentration with insulin, it is not metabolized by the liver and does not undergo any extraction during the first hepatic passage [16]. Therefore, it can be indirectly measured to access insulin secretion [16]. Finally, we used an HOMA-IR like index to access insulin sensitivity instead of the hyperinsulinemic euglycemic clamp. Which is the reference method, though very demanding and time-consuming [25]. Nonetheless, several indices including the HOMA-IR have been developed to facilitate this assessment. It is true that Sobngwi and co-workers demonstrated that fasting insulin sensitivity indices like HOMA-IR are modest predictors of insulin sensitivity measured by euglycemic clamp in non-diabetes African subjects [26], Noteworthy, Bonora and co-workers bolstered a strong correlation between the HOMA-IR and the hyperinsulinemic euglycemic clamp in the general population, which enabled them to recommend using the HOMA-IR in conditions where realization of the clamp method is not easy [27]. Moreover, to the best of our knowledge, there is no a study which determined the HOMA-IR threshold or the estimated percentile to define insulin resistance in African population; therefore, we used a threshold which was non-specific to our study population. Notwithstanding these limitations, our sample size was relatively high and we used rigorous methodological and statistical procedures to examine our research questions.

## Conclusion

Insulin resistance may be highly prevalent in the black African HIV-infected population, perhaps mainly driven by obesity. This calls for the implementation or strengthening of obesity prevention programs in the HIV-infected population in other to prevent or postpone the occurrence of insulin resistance and related complications.
